# Impact of a nutrition and physical activity intervention (ENRICH: Exercise and Nutrition Routine Improving Cancer Health) on health behaviors of cancer survivors and carers: a pragmatic randomized controlled trial

**DOI:** 10.1186/s12885-015-1775-y

**Published:** 2015-10-15

**Authors:** E. L. James, F. G. Stacey, K. Chapman, A. W. Boyes, T. Burrows, A. Girgis, G. Asprey, A. Bisquera, D. R. Lubans

**Affiliations:** 1 School of Medicine and Public Health, Priority Research Centre for Health Behavior, Priority Research Centre in Physical Activity and Nutrition, The University of Newcastle, Callaghan, NSW Australia; 2 Hunter Medical Research Institute, New Lambton Heights, NSW Australia; 3 Cancer Council New South Wales, Woolloomooloo, NSW Australia; 4 Priority Research Centre for Health Behavior, School of Medicine and Public Health, The University of Newcastle, Callaghan, NSW Australia; 5 School of Health Sciences, Priority Research Centre in Physical Activity and Nutrition, The University of Newcastle, Callaghan, NSW Australia; 6 Centre for Oncology Education and Research Translation (CONCERT), Ingham Institute for Applied Medical Research, UNSW Medicine, Liverpool, NSW Australia; 7 School of Education, Priority Research Centre in Physical Activity and Nutrition, The University of Newcastle, Callaghan, NSW Australia

**Keywords:** Cancer, Physical activity, Nutrition, Randomized controlled trial, Health behavior, Carer

## Abstract

**Background:**

Physical activity and consuming a healthy diet have clear benefits to the physical and psychosocial health of cancer survivors, with guidelines recognising the importance of these behaviors for cancer survivors. Interventions to promote physical activity and improve dietary behaviors among cancer survivors and carers are needed. The aim of this study was to determine the effects of a group-based, face-to-face multiple health behavior change intervention on behavioral outcomes among cancer survivors of mixed diagnoses and carers.

**Methods:**

The Exercise and Nutrition Routine Improving Cancer Health (ENRICH) intervention was evaluated using a two-group pragmatic randomized controlled trial. Cancer survivors and carers (*n* = 174) were randomly allocated to the face-to-face, group-based intervention (six, theory-based two-hour sessions delivered over 8 weeks targeting healthy eating and physical activity [PA]) or wait-list control (after completion of 20-week data collection). Assessment of the primary outcome (pedometer-assessed mean daily step counts) and secondary outcomes (diet and alcohol intake [Food Frequency Questionnaire], self-reported PA, weight, body mass index, and waist circumference) were assessed at baseline, 8-and 20-weeks.

**Results:**

There was a significant difference between the change over time in the intervention group and the control group. At 20 weeks, the intervention group had increased by 478 steps, and the control group had decreased by 1282 steps; this represented an adjusted mean difference of 1761 steps (184 to 3337; *P* = 0.0028). Significant intervention effects for secondary outcomes, included a half serving increase in vegetable intake (difference 39 g/day; 95 % CI: 12 to 67; *P* = 0.02), weight loss (kg) (difference -1.5 kg; 95 % CI, -2.6 to -0.3; *P* = 0.014) and change in body mass index (kg/m^2^) (difference -0.55 kg/m^2^; 95 % CI, -0.97 to -0.13; *P* = 0.012). No significant intervention effects were found for self-reported PA, total sitting time, waist circumference, fruit, energy, fibre, alcohol, meat, or fat consumption.

**Conclusions:**

The ENRICH intervention was effective for improving PA, weight, body mass index, and vegetable consumption even with the inclusion of multiple cancer types and carers. As an example of successful research translation, the Cancer Council NSW has subsequently adopted ENRICH as a state-wide program.

**Trial registration:**

Australian New Zealand Clinical Trials Register identifier: ANZCTRN1260901086257.

## Background

Consuming a healthy diet and participating in physical activity (PA) has been shown to enhance general physical and psychosocial health in cancer survivors and reduce risk of recurrence, cancer-specific and all-cause mortality [1–9]. Despite the potential benefits of healthy lifestyle behaviors, and international guidelines for survivors [10–14], survivors’ behaviors remain similar to the general population [15–19], with few meeting the recommendations (e.g., only 5 % of survivors meeting the three recommendations for PA, fruit/vegetables and non-smoking) [15]. Despite the challenges that survivors face throughout diagnosis and treatment, they can be motivated to make behavioral improvements and report being interested in behavior change programs [20, 21]. Carers of cancer survivors share many of the same behavioral risk factors [22, 23] as survivors, and also experience poor physical and psychosocial health [24]. Inclusion of carers and survivors together in interventions can result in improvements in well-being, social support, diet and PA behavior for both the cancer survivor and their carer [24, 25].

Health behaviors are inter-related in terms of the psychological, social, and environmental factors that reinforce them, and multiple unhealthy behaviors often co-exist [26]. Diet and PA behaviors in particular, are closely related, and evidence suggests that interventions targeting both behaviors simultaneously offer the most promise for sustained behavior change [27, 28]. In the existing climate of limited resources, programs that are appropriate for survivors of multiple cancer types are appealing (as opposed to offering several different behavior change programs for each specific cancer type).

Previous trials have reported that cancer survivors can safely undertake both supervised and unsupervised PA interventions during and after cancer treatment [9, 29–32]. PA guidelines for cancer survivors encompass individual behaviors, which are all independent risk factors, relating to reduction of sitting time, and undertaking both aerobic and resistance activity [12–14]. However, there are some gaps in the PA research. Few trials have tested a resistance training intervention; or used objective measures of PA; or assessed behavior change after the intervention [9, 31, 33, 34]. In addition, most trials targeting PA and/or diet intervention have been aimed at breast cancer survivors. Previous diet interventions have been delivered as part of multiple health behavior interventions, using a range of delivery modes over a period of 6 to 12 months, and found modest improvements in fruit and vegetable consumption and lower fat intake [35, 36]. One multiple health behavior intervention targeted both breast and prostate cancer survivors, using a 10-month tailored print intervention, and reported significant improvements to exercise behavior, fruit, vegetables, and lower fat intake [29]. These data are promising and demonstrate the feasibility of recruiting and retaining cancer survivors into efficacious multiple health behavior programs. To our knowledge, there are no trials that have included survivors of any cancer type together with their carers.

With increasing numbers of survivors, more research is needed on the most efficient and efficacious ways to support their behavior change. We partnered with a major cancer charity to develop an intervention that could be implemented routinely. This intervention meets definition criteria for a pragmatic trial as program delivery and recruitment was managed by the cancer charity, program eligibility was broad, the goals of the intervention were applied flexibly based on the preferences of the participant, and the program outcomes are directly relevant to funders and the community [37]. The aim of this paper is to report the effects of a theory-based, group-delivered, face-to-face multiple health behavior change intervention (ENRICH) on behavioral outcomes among a mixed group of cancer survivors and carers.

## Methods

**Fig. 1:**
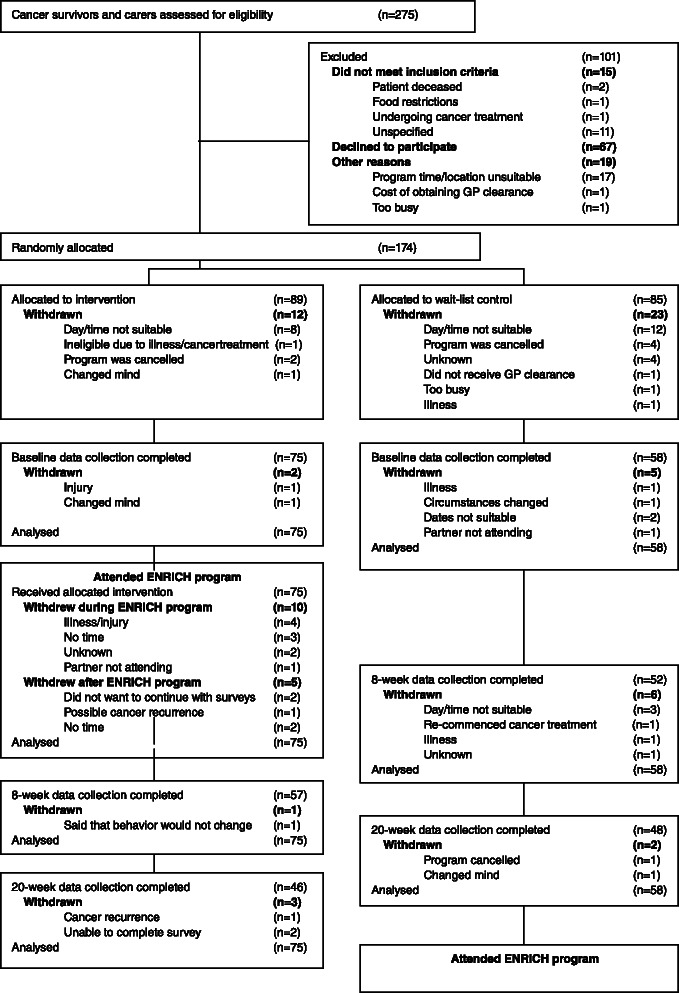
Participant flow diagram

A two-arm pragmatic randomized controlled trial (RCT) with a wait-list control group (who attended the intervention program after completing 20 week data collection) was conducted. The study protocol is described in detail elsewhere [38]. In brief, participants completed assessments at baseline, 8 weeks (intervention completion), and 20 weeks (Fig. 1). The primary outcome was pedometer-assessed step counts at 20 weeks post-baseline (i.e., 3 months after completion of the intervention). Secondary outcomes included: self-reported PA and resistance training, sitting time, dietary intake, weight, and body mass index (BMI). While weight management is not a lifestyle behavior, it is the key target of lifestyle behavior strategies [39].

### Eligibility

Eligibility criteria included: 1) individual diagnosed with cancer who had completed all active cancer treatment (“cancer survivor”) or “carer” of cancer survivor; 2) no food restrictions as a result of surgery or treatment; 3) aged 18 years or older; 4) fluent in English; 5) signed medical clearance from their General Practitioner; and 6) with a functional performance score of two or less on the Eastern Cooperative Oncology Group criteria (that is “at least ambulatory and capable of all self-care but unable to carry out any work activities or up and about more than 50 % of waking hours”) [40].

### Participant recruitment

The trial was approved by the Human Research Ethics Committee of the University of Newcastle (H-2009-0347), and was registered with the Australian New Zealand Clinical Trials Register (ANZCTRN1260901086257). The method of recruitment was designed to closely align with how ‘real world’ recruitment would occur if ENRICH were a community-based program offered across Australia. Therefore, participants were recruited by referrals from health professionals, medical centers, community health centers, cancer support groups, local media, and various Cancer Council NSW resources (website, mailing lists, and publications). Participants provided written informed consent to participate, and obtained signed medical clearance from their General Practitioner. Cancer survivors and carers could participate independently or together. Participation was not dependent on both members of the dyad consenting (i.e., survivors could participate without their carer participating and vice versa). The trial did not aim to recruit cancer survivors and carers together as a dyad.

### Random assignment

Consenting participants were stratified by age and gender and were randomly assigned by the Project Co-ordinator using a random number table to either an immediate program (within one month of consent) (intervention) or wait-list program (occurring 6–8 months after consent).

### Study conditions

Intervention: Four weekly, 2-h sessions, and two 2-h fortnightly sessions were provided (total 6 sessions). The gradual lengthening of time between sessions was designed to promote self-management strategies and encourage maintenance of behaviors. Participants were provided with a workbook (which contained program notes, activities, and handouts), open pedometer and Gymstick™ (a lightweight graphite shaft, with elastic tubing and foot straps that provide resistance to exercise all major muscle groups). Gymsticks™ have been found to be acceptable and effective in improving muscular fitness in a trial with sedentary older adults [41]. Each group-based session delivered simultaneous multiple health behavior content covering a home-based walking program (using a pedometer), home-based resistance training program (using a Gymstick™), and information about healthy eating (the Australian Guide to Healthy Eating, fruit and vegetables, maintaining a healthy weight, fats, meat, salt, dietary supplements, alcohol, and food labels). Sessions included a mix of didactic information delivery (guidelines and recommendations, strategies to increase PA and healthy foods, overcoming barriers, food budgeting) and practical activities (e.g., label reading, recipe modification, demonstration and practice of resistance exercises, setting step goals for the home-based walking program). To encourage maintenance of behavior change, at the final session participants received information about other community-based programs and support services. Recommended behavior changes were based on current guidelines [10–14] with participants encouraged to reflect on personal areas for improvement and select key behaviors to change.

Each session was co-facilitated by a qualified exercise specialist (Accredited Exercise Physiologist or Physiotherapist) and an Accredited Practising Dietician. Facilitators attended study-specific training and were provided with a handbook, session guides, and program resources. The content and delivery of sessions was operationalized using the principles of Social Cognitive Theory [42] and a chronic disease self-management framework [43]. The specific behavior change strategies that were operationalized included goal setting, self-monitoring, self-efficacy, outcome expectancies, barriers and facilitators, and social support.

Control: Participants attended the 8-week, 6-session ENRICH program after completing 20-week study measures.

### Measurement

Data were collected by pen-and-paper mailed survey (demographics, physical activity, sitting time, dietary behaviors, weight, height, waist circumference), and sealed pedometer and pedometer log sheet.

#### Primary outcome

The primary outcome was step counts at 20 weeks post-baseline, measured by a sealed (Yamax SW200) pedometer (the sealed pedometers used for data collection were different to the open pedometers provided to participants as part of the intervention). As pedometers were sealed, the variation of steps between days was not captured. Participants recorded the time they put on and removed the pedometer each day. Previous studies have reported a significant correlation between pedometer wear time and steps [44]. To establish mean daily step count, total steps were divided by the number of days with wear time greater than five hours, and 0.5 for each day where pedometer wear time was less than five hours. Pedometers are small, relatively inexpensive devices worn at the hip to count number of steps walked per day, and they have been shown to have good reliability and validity [45, 46]. Participants also completed a log sheet to record other PA such as resistance training, swimming, water aerobics, and cycling that were not captured by pedometry, which is important to assess change in PA due to the intervention [47]. These activities were converted to sex-specific step counts using the values reported elsewhere [47] (Table 2), and were added to the total step count value. A methodological secondary aim was to explore the feasibility and usefulness of pedometer diaries to record key behaviors (e.g., resistance activities) that are not captured by pedometry. Analysis of step counts using both the raw pedometer data, and imputed step count data were assessed separately.

#### Secondary outcomes

Participants self-reported their weight, height, and waist circumference (using standardized instructions) [48, 49].

The frequency and duration of PA was measured with the Active Australia survey [50], plus two purpose-designed questions about resistance training. The mean number of minutes of walking, resistance training, and moderate-to-vigorous PA reported over the past week were calculated, and vigorous activity was double-weighted to account for additional energy expenditure [50].

Sedentary behavior was assessed with five items asking about time spent sitting in the last working and non-working day across five domains [51]. Total sitting time on last working day and non-working day was computed by adding time spent sitting in each domain.

Dietary intake was assessed using the 74-item Dietary Questionnaire for Epidemiological Studies version 2 food frequency questionnaire (FFQ) [52–54]. The average daily amount of foods from food groups that were specifically targeted in the intervention and of relevance to cancer survivors were calculated, including fruit (g/day), vegetables (g/day), red meat (g/day), processed meat (g/day), dietary fibre (g/1000 kJ) and alcohol intake (g/day and percent of daily energy). Serves of fruits (total fruit excluding fruit juices) and vegetables (total vegetables including potato) were calculated by summing the weight of food items in the FFQ coded as fruits or vegetables and dividing by the serve size reported in the Australian Guide to Healthy Eating (fruits, 150 g and vegetables, 75 g). Nutrient intakes were computed from the food composition database of Australian foods, NUTTAB 1995 [55].

### Intervention adherence and program satisfaction

The program co-ordinator attended each ENRICH session to assess facilitator compliance with the ENRICH program. Each program facilitator completed a 1-page assessment after each ENRICH session to identify any issues with the session objectives and content, resources, location and equipment, participants, and timing and questions. At the final ENRICH session, participants also completed an evaluation form that assessed their satisfaction with the program.

### Statistical analysis

Descriptive statistics are presented as mean (+/- standard deviation) for continuous variables and as number and percent for categorical variables. A repeated measures analysis was conducted using linear mixed models in IBM SPSS Statistics 21 [56], with the random statement to fit a random intercept model. The primary outcome in the model was mean daily step count, computed by dividing the total pedometer steps recorded by the number of days worn (wear time greater than 5 h equalled 1 day; wear time of 5 h or less equalled ½ day). The predictor variables included treatment, time and the interaction of treatment-by-time. The coefficient of the interaction term was used to determine if there was a difference in the trends in step counts over time between participants in the different treatment groups. We accounted for clustering of cancer survivor and carer dyads in the model. However, as the addition of a cluster variable made no difference to the standard errors of the coefficients or model fit statistics, it was removed from the final model (with cluster: AIC 5523.7, BIC 5517.7; without cluster: AIC 5523.6, BIC 5529.4; ICC 0.28). Differences in least squares means with 95 % confidence intervals and the group by time *p*-value are presented.

Subgroup analyses were undertaken to explore whether the intervention effect varied for: participants who were meeting/not meeting the recommended number of fruit servings (less than 2 serves/day); vegetarians/those who had consumed red and processed meat; participants who reported consuming alcohol/non-drinkers; participants with a BMI greater than 25 kg/m^2^ (overweight or obese) at baseline versus participants whose BMI was less than 25 kg/m^2^ (underweight/healthy weight); and for the sample of cancer survivors separately.

### Sample size

Forty-two subjects per group were required to detect a mean difference of 2000 steps per day in pedometer-assessed step counts with 80 % power and 5 % significance, with a standard deviation of 3200 steps. The effect size estimate of 2000 steps per day change was based on a clinically meaningful difference [45]. To ensure adequate sample size for secondary outcomes and to account for attrition and missing data, we aimed to recruit 75 subjects per group.

## Results

### Participants

Two-hundred and seventy-five potential participants expressed interest and were screened for eligibility by the Project Co-ordinator over the telephone. One-hundred and seventy-four participants were randomized and 133 completed baseline data collection (Fig. 1). In order to provide a consistent time reference, participants completed baseline one week prior to the first ENRICH program session. The majority of participants who withdrew, did so prior to attending any ENRICH sessions (*n* = 51). At 8-week data collection, 76 % (*n* = 57) of intervention participants and 89.7 % (*n* = 52) of control participants were retained. At 20-weeks, 61.3 % (*n* = 46) of the intervention group, and 82.8 % (*n* = 48) of the control group were retained.

### Baseline characteristics

Groups had similar baseline demographic characteristics (Table 1), except that intervention participants were more likely to have received chemotherapy treatment for their cancer and to have been diagnosed with arthritis. As this is a randomized trial, these differences were the result of chance [57]. There were no significant differences between those who dropped out and those who completed follow-ups on key demographic characteristics (gender, age, marital status, employment, education, income, or cancer survivor/carer status).

**Table 1:** Baseline characteristics of participants (n = 133)

	Control (*n* = 58)			Intervention (*n* = 75)		
Characteristic	N	% (of responses)	Sample size	N	% (of responses)	Sample size
Age, years, Mean (SD)	58.1 (11.2)		57	56.2 (12.6)		75
Female gender	43	74.1	58	60	80.0	75
Married/de facto	38	66.7	57	55	73.3	75
Completed post-school qualifications	41	71.9	57	54	73.0	74
Employed (full-time or part-time)	26	45.6	57	34	45.9	74
Weekly family income			56			75
-Less than $499	11	19.6		12	16.0	
-$500-$1000	14	25.0		16	21.3	
-More than $1000	14	25.0		25	33.3	
-Prefer not to answer	17	30.4		22	29.3	
Current smoker	3	5.3	57	3	4.2	72
Number of co-morbidities (ever had OR have)			58			75
-0	16	27.6		13	17.3	
-1–3	33	56.9		50	66.7	
-4 or more	9	15.5		12	16.0	
Types of co-morbidities^a^						
-Musculoskeletal disorders	20	37.7	53	28	38.4	73
-Mental health problems	15	27.8	54	27	36.5	74
-Arthritis	16	28.6	56	34	45.9	74
-High blood pressure	18	32.1	56	18	24.3	74
-High cholesterol	19	33.9	56	25	33.3	75
-Chronic headache/migraine	9	16.1	56	7	9.5	74
-Lung conditions	7	12.5	56	13	17.6	74
-Heart condition	4	7.1	56	5	6.8	74
-Stroke	2	3.6	56	0	0	74
-Diabetes	4	7.1	56	2	2.7	73
-Stomach ulcer	3	5.4	56	2	2.7	74
Cancer survivor	43	75.4	57	53	70.7	75
Carer	9	15.8	57	15	20.0	75
Both cancer survivor and carer	5	8.8	57	7	9.3	75
Relationship to cancer survivor:			14			19
-Spouse/partner	11	78.6		12	63.2	
-other relative or friend	3	21.4		7	36.8	
Cancer type^a^			48			60
-Bowel/colorectal	5	10.4		3	5.0	
-Breast	28	58.3		36	60.0	
-Prostate	7	14.6		7	11.7	
-Melanoma	3	6.3		4	6.7	
-Other (eg. non-Hodgkins lymphoma, Leukaemia, ovarian, thyroid)	13	27.1		17	28.3	
Time since diagnosis, months, Mean (SD)	45.2 (52.3)		47	39.3 (56.7)		57
Treatment received (EVER)^a^						
-Surgery	45	93.8	48	55	93.2	59
-Chemotherapy	28	62.2	45	45	84.9	53
-Radiotherapy	30	63.8	47	32	68.1	47
-Hormone treatment	20	48.8	41	30	66.7	45
Cancer in remission	36	80.0	45	44	77.2	57
Number of cancer diagnoses			48			60
-1	43	89.6		54	90.0	
-2	4	8.3		5	8.3	
-3 or more	1	2.1		1	1.7	

^a^Participants could select more than one response, so the percentage may add up to more than 100 %

Participants in both groups reported similar PA behaviors at baseline (Table 2) and some small differences between groups on dietary behaviors. The control group reported higher energy consumption (by 399 kJ), total fat (by 6 g), saturated fat (by 1.7 g), and red meat consumption (by 16.7 g).

**Table 2:** Baseline health behaviors (*n* = 133)

	Control (*n* = 58)		Intervention (*n* = 75)	
Characteristic	Mean (SD)	Sample size	Mean (SD)	Sample size
Physical activity				
Raw step counts (per day)	8090.7 (3298.8)	50	7850.0 (3111.5)	68
Moderate-to-vigorous PA (minutes per week)	108.7 (187.1)	55	82.9 (91.9)	69
Resistance training (minutes per week)	13.5 (26.1)	57	14.7 (39.3)	74
Sedentary behavior				
Total sitting time on last work day (minutes per day)	547.5 (235.8)	17	774.8 (840.0)	22
Total sitting time on last non-work day (minutes per day)	519.0 (407.8)	23	522.2 (240.9)	33
Dietary behavior (per day)		55		73
Fruit (g)	254.6 (144.6)		222.3 (117.1)	
Fruit (serves)^a^	1.7 (1.0)		1.5 (0.8)	
Vegetables (g)	168.7 (81.0)		174.8 (95.5)	
Vegetables (serves)^b^	2.3 (1.1)		2.3 (1.3)	
Fibre grams per 1000 kJ	3.2 (0.8)		3.3 (0.9)	
Fibre (g)	21.4 (8.4)		21.0 (7.0)	
Energy (kJ)	6853 (2495)		6454 (1826)	
Total fat (g)	69.4 (30.2)		63.4 (21.2)	
Saturated fat (g)	26.6 (12.8)		24.9 (9.7)	
Red meat (g)^c^	66.2 (88.1)		49.5 (42.4)	
Processed meat (g)	17.0 (27.4)		12.2 (13.5)	
% energy from alcohol (%)	4.7 (5.8)		5.1 (7.9)	
Alcohol (g)^d^	10.1 (14.4)		9.5 (13.5)	
Body composition				
Weight (kg)	76.2 (17.8)	56	72.8 (14.6)	73
BMI (kg/m^2^)	27.5 (6.1)	54	26.7 (5.2)	73
Waist circumference (cm)	93.9 (14.0)	50	91.9 (16.2)	63

^a^1 serve = 150 g

^b^1 serve = 75 g

^c^1 serve = 65 g cooked lean red meat

^d^1 standard drink = 10 g alcohol in Australia

### Intervention adherence and program satisfaction

All intervention session components were delivered by program facilitators. The majority of intervention participants (76 %; *n* = 57) attended at least five of the six ENRICH face-to-face sessions. The mean number of participants in each ENRICH group program was 10. At completion of the program, participants agreed that (1 = strongly disagree to 4 = strongly agree): they trusted the information provided as part of the program $\left(\overline{\mathrm{x}} = 3.8\right)$; participation was worth their time and effort $\left(\overline{\mathrm{x}}=3.7\right)$; course leaders were organized $\left(\overline{\mathrm{x}}=3.8\right)$ and managed the topics well $\left(\overline{\mathrm{x}}=3.7\right)$; the program attendees worked well together $\left(\overline{\mathrm{x}}=3.6\right)$; and everyone had a chance to speak $\left(\overline{\mathrm{x}}=3.8\right)$.

### Primary outcome: Pedometer-assessed PA

There was evidence of a change in mean daily step counts over time between intervention and control at 8 weeks (adjusted mean difference from baseline 2095 steps/day; 95 % CI: 909 to 3281) that was maintained at 20 weeks (mean difference from baseline 1761 steps/day; 95 % CI: 184 to 3338) (*P* = 0.0028) (Table 3). The difference consisted of the control group decreasing step counts by 1294 and the intervention increasing steps by 800 steps at 8 weeks. This effect was amplified after accounting for ‘other’ activities and imputing equivalent step values for cycling, swimming, water aerobics and resistance training. The mean difference of the change over time between the groups at 8 weeks was 2810 steps/day (95 % CI: 1238 to 4382) and at 20 weeks was 2782 steps/day (95 % CI: 818 to 4745) (*P* = 0.0009).

**Table 3:** Mean difference in the physical activity and sedentary behavior outcomes from baseline to 8 weeks and 20 weeks, and *p* value for the difference in change between treatment groups

		Mean change from baseline (95 % CI)		Adjusted mean difference (95 % CI)	Group-by-time
Outcome	Time from baseline	Control	Intervention		*P*-value
Mean daily steps (pedometer-assessed)	8 week	−1294 (-2214 to -374.1)	800.8 (52.3 to 1549.3)	2094.7 (908.9 to 3280.5)	0.0028
	20 week	−1282 (-2394 to -170.6)	478.8 (-639.4 to 1597.0)	1761.0 (184.3 to 3337.8)	
Mean daily steps (with imputation of steps for swimming, cycling, resistance training)	8 week	−1672 (-2873 to -471.9)	1137.8 (122.3 to 2153.3)	2810.1 (1237.8 to 4382.3)	0.0009
	20 week	−2124 (-3546 to -702.9)	657.20 (-697.1 to 2011.5)	2781.5 (818.2 to 4744.8)	
Moderate-to-vigorous PA (minutes per week)	8 week	9.6 (-26.2 to 45.4)	33.87 (-4.7 to 72.4)	24.3 (-28.3 to 76.8)	0.2168
	20 week	8.7 (-21.4 to 38.9)	−16.2 (-39.8 to 7.5)	−24.9 (-63.2 to 13.4)	
Resistance training (minutes per week)	8 week	16.0 (-0.01 to 32.0)	38.3 (21.2 to 55.5)	22.3 (-1.1 to 45.8)	0.1039
	20 week	12.3 (-8.9 to 33.5)	29.3 (15.6 to 43.0)	17.0 (-8.3 to 42.2)	
Total sitting time on last WORK DAY, excluding sleep (minutes per day):	8 week	201.3 (-131.3 to 534.0)	−132.5 (-347.0 to 82.1)	−333.8 (-729.6 to 62.0)	0.2412
	20 week	162.9 (-41.4 to 367.2)	−28.5 (-271.5 to 214.4)	−191.4 (-508.8 to 126.0)	
Total sitting time on last NON-WORK DAY, excluding sleep (minutes per day):	8 week	82.6 (-140.6 to 305.8)	69.5 (-99.1 to 238.1)	−13.1 (-292.8 to 266.6)	0.4275
	20 week	−74.7 (-231.6 to 82.2)	52.4 (-84.7 to 189.5)	127.1 (-81.3 to 335.4)	

### Secondary outcomes

There were no significant group-by-time effects for weekly minutes of moderate-to-vigorous PA, resistance training, or minutes per day of sitting time (Table 3). There was a significant difference in the change over time between intervention and control for daily vegetable consumption at 8 weeks (mean 24 g; 95 % CI: -0.9 to 49) and 20 weeks (mean 39 g; 95 % CI: 12 to 67) (*P* = 0.019), which equates to a difference of 0.3 to 0.5 of a serve. Both groups reported increased fruit and fibre consumption, decreased alcohol consumption, and fat intake (Table 4). However, these differences in the change over time between intervention and control group were not significant.

**Table 4:** Mean difference in the diet and body composition outcomes from baseline to 8 weeks and 20 weeks, and *p* value for the difference in change between treatment groups

		Mean change from baseline (95 % CI)		Adjusted mean difference (95 % CI)	Group-by-time
Outcome	Time from baseline	Control	Intervention		*P*-value
Fruit (excluding juice) (g/day)	8 week	7.3 (-27.9 to 42.5)	36.9 (-0.3 to 74.1)	29.6 (-21.6 to 80.8)	0.3793
	20 week	12.9 (-17.0 to 42.9)	50.9 (1.2 to 100.5)	38.0 (-20.0 to 96.0)	
Vegetables (g/day)	8 week	−0.5 (-19.1 to 18.1)	23.6 (6.9 to 40.2)	24.1 (-0.9 to 49.0)	0.0188
	20 week	−7.1 (-26.9 to 12.7)	32.4 (13.3 to 51.4)	39.4 (12.0 to 66.9)	
Vegetables (serves/day)	8 week	−0.01 (-0.3 to 0.2)	0.3 (0.1 to 0.5)	0.3 (-0.01 to 0.7)	0.0188
	20 week	−0.1 (-0.4 to 0.2)	0.4 (0.2 to 0.7)	0.5 (0.2 to 0.9)	
Dietary fibre (g/1000 kJ)	8 week	0.1 (-0.01 to 0.3)	0.3 (0.2 to 0.5)	0.2 (-0.02 to 0.4)	0.1942
	20 week	0.1 (-0.1 to 0.2)	0.2 (0.1 to 0.4)	0.2 (-0.1 to 0.4)	
Energy (kJ/day)	8 week	−244 (-806 to 318)	−492 (-912 to -72)	−248 (-949 to 453)	0.5739
	20 week	111 (-836 to 1057)	−436 (-881 to 9)	−547 (-1592 to 499)	
Total fat (g/day)	8 week	−3.4 (-10.3 to 3.5)	−7.4 (-12.0 to -2.9)	−4.0 (-12.3 to 4.2)	0.4165
	20 week	0.5 (-10. 6 to 11.5)	−7.1 (-11.7 to -2.6)	−7.6 (-19.5 to 4.4)	
Saturated fat (g/day)	8 week	−1.5 (-4.1 to 1.1)	−3.7 (-5.5 to -1.9)	−2.2 (-5.4 to 0.9)	0.2827
	20 week	−0.4 (-4.4 to 3.6)	−3.4 (-5.3 to -1.5)	−3.0 (-7.4 to 1.4)	
Red meat (g/day)	8 week	1.2 (-20.6 to 23.1)	−2.9 (-13.3 to 7.5)	−4.1 (-28.3 to 20.1)	0.4208
	20 week	−6.0 (-28.3 to 16.3)	0.8 (-8.2 to 9.8)	6.8 (-17.3 to 30.9)	
Processed meat (g/day)	8 week	−1.7 (-5.8 to 2.4)	0.1 (-2.7 to 2.9)	1.8 (-3.2 to 6.7)	0.6659
	20 week	−2.6 (-8.9 to 3.6)	0.5 (-2.7 to 3.6)	3.1 (-3.9 to 10.1)	
Alcohol (g/day)	8 week	−0.4 (-1.8 to 1.1)	−2.2 (-4.5 to 0.1)	−1.8 (-4.5 to 0.9)	0.2331
	20 week	−1.6 (-3.6 to 0.5)	−1.3 (-4.0 to 1.3)	0.2 (-3.1 to 3.6)	
% of energy provided by alcohol (%)	8 week	−0.3 (-1.2 to 0.6)	−1.0 (-2.0 to 0.1)	−0.7 (-2.1 to 0.7)	0.4265
	20 week	−0.5 (-1.6 to 0.6)	−0.2 (-1.7 to 1.3)	0.3 (-1.6 to 2.1)	
Weight (kgs)	8 week	0.04 (-0.5 to 0.6)	−1.4 (-2.3 to -0.4)	−1.4 (-2.5 to -0.3)	0.0140
	20 week	−0.1 (-0.8 to 0.6)	−1.6 (-2.5 to -0.7)	−1.5 (-2.6 to -0.3)	
BMI (kg/m^2^)	8 week	0.02 (-0.2 to 0.2)	−0.5 (-0.9 to -0.1)	−0.5 (-1.0 to -0.1)	0.0120
	20 week	−0.02 (-0.3 to 0.2)	−0.6 (-0.9 to -0.2)	−0.6 (-1.0 to -0.1)	
Waist circumference (cm)	8 week	−1.5 (-3.8 to 0.7)	−3.8 (-5.9 to -1.7)	−2.3 (-5.4 to 0.7)	0.2361
	20 week	−2.1 (-4.0 to -0.2)	−2.5 (-5.8 to 0.8)	−0.4 (-4.3 to 3.4)	

Intervention participants reported weight loss at 8 weeks, with an adjusted mean difference of -1.4 kg (95 % CI: -2.5 to -0.3) compared to the change in control. At 20 weeks, the difference remained significant (mean -1.5 kg; 95 % CI: -2.6 to -0.3) (*P* = 0.014). For intervention participants, this decrease equated to an average 1.9 % reduction in body weight from baseline to 8 weeks, and 2.2 % reduction in body weight from baseline to 20 weeks. For body mass index, the mean difference at 8 weeks was -0.5 kg/m^2^ (95 % CI: -0.98 to -0.11) and -0.55 kg/m^2^ (95 % CI: -0.97 to -0.13) at 20 weeks (*P* = 0.012). Both groups decreased waist circumference, however there was no difference in the change over time between the intervention and control groups (*P* = 0.236).

### Subgroup and sensitivity analyses

Participants in both study groups who consumed less than two serves of fruit per day at baseline (*n* = 93), reported non-significant increases to daily serves of fruit. There was no evidence of an intervention effect for participants who reported consuming red or processed meat (*n* = 123-126; *P* = 0.4 to 0.6), or those that had consumed alcohol (*n* = 126; *P* = 0.2).

Participants were divided into two sub-groups based on BMI category, and whether weight loss would be considered a positive outcome. Control participants who were overweight (*n* = 50) or obese (*n* = 30) (BMI > 25 kg/m^2^) at baseline decreased their mean daily steps at 8 weeks (-1370; 95 % CI: -2722 to -18.1) and compared to intervention participants who remain unchanged from baseline (*P* = 0.0349). Among participants whose BMI was less than 25 kg/m^2^ (underweight *n* = 3; healthy weight *n* = 44) at baseline, there was no intervention effect on step counts at 8 weeks (mean difference 210; 95 % CI: -787 to 1206) or 20 weeks (mean difference -52; 95 % CI: -1711 to 1607) (*P* = 0.1). Among participants whose BMI was greater than 25 kg/m^2^, there was a significant group-by-time effect for weight at 8 weeks (adjusted mean difference of -2.2 kg; 95 % CI: -3.9 to -0.5) and 20 weeks (-2.0 kg; 95 % CI: -3.7 to -0.4) (*P* = 0.0157). At 8 weeks, the adjusted mean difference for BMI was -0.8 kg/m^2^ (95 % CI: -1.5 to -0.2) and at 20 weeks was -0.7 kg/m^2^ (95 % CI: -1.3 to -0.1) (*P* = 0.0181). Overweight/obese participants in both groups reported reductions in waist circumference, with the adjusted mean difference at 8 weeks of -3.3 cm (95 % CI: -7.0 to 0.4) and 20 weeks of 0.2 cm (95 % CI: -5.6 to 6.0) (*P* = 0.0722). There was no group-by-time intervention effect for participants whose BMI was lower than 25 kg/m^2^ at baseline, on waist circumference, BMI, or weight (Table 5).

**Table 5:** Subgroup and sensitivity analyses

		Mean change from baseline (95 % CI)			
Outcome (Subgroup)	Time from baseline	Control	Intervention	Difference (Intervention - Control)	Time x Group *P*-value
Overweight and obese at baseline (n = 80; BMI >25 kg/m^2^)					
Mean daily steps	8 week	−1370 (-2722 to -18.1)	960.1 (-158.5 to 2078.8)	1793.6 (131.5 to 3455.7)	0.0349
	20 week	−1366 (-2903 to 170.7)	768.0 (-751.7 to 2287.7)	1597.2 (-267.8 to 3462.2)	
BMI (kg/m^2^)	8 week	0.03 (-0.2 to 0.3)	−0.8 (-1.4 to -0.2)	−0.8 (-1.5 to -0.2)	0.0181
	20 week	−0.01 (-0.4 to 0.3)	−0.7 (-1.2 to -0.3)	−0.7 (-1.3 to -0.1)	
Weight (kgs)	8 week	0.06 (-0.6 to 0.8)	−2.1 (-3.7 to -0.6)	−2.2 (-3.9 to -0.5)	0.0157
	20 week	−0.06 (-1.0 to 0.8)	−2.1 (-3.5 to -0.7)	−2.0 (-3.7 to -0.4)	
Waist circumference (cms)	8 week	−1.0 (-2.5 to 0.5)	−4.3 (-7.7 to -0.9)	−3.3 (-7.0 to 0.4)	0.0722
	20 week	−2.6 (-5.0 to -0.2)	−2.4 (-7.7 to 2.9)	0.2 (-5.6 to 6.0)	
Normal and underweight at baseline (*n* = 47; BMI < 25 kg/m^2^)					
Mean daily steps	8 week	−1312 (-2343 to -280.4)	209.7 (-786.9 to 1206.2)	1521.6 (87.3 to 2955.8)	0.1046
	20 week	−1271 (-3018 to 476.)	−51.7 (-1711 to 1607.3)	1219.6 (-1190 to 3628.9)	
BMI (kg/m^2^)	8 week	0.02 (-0.3 to 0.3)	−0.1 (-0.3 to 0.1)	−0.1 (-0.5, 0.3)	0.7127
	20 week	−0.05 (-0.5 to 0.4)	−0.2 (-0.5 to -0.02)	−0.2 (-0.7 to 0.3)	
Weight (kgs)	8 week	−0.02 (-0.9 to 0.9)	−0.2 (-0.7 to 0.3)	−0.2 (-1.2 to 0.8)	0.8127
	20 week	−0.2 (-1.5 to 1.0)	−0.7 (-1.2 to -0.1)	−0.4 (-1.8 to 1.0)	
Waist circumference (cms)	8 week	−2.4 (-7.4 to 2.6)	−2.9 (-4.8 to -1.1)	−0.5 (-5.9 to 4.8)	0.7309
	20 week	−1.2 (-3.9 to 1.5)	−2.3 (-4.2 to -0.4)	−1.2 (-4.4 to 2.1)	
Other subgroups					
Processed meat (g/day) (consuming any processed meat; n = 123)	8 week	−1.8 (-6.3 to 2.7)	0.3 (-2.9 to 3.4)	−3.3 (-10.6 to 4.0)	0.6405
	20 week	−2.8 (-9.8 to 4.2)	0.8 (-2.8 to 4.3)	−1.7 (-8.0 to 4.6)	
Red meat (g/day) (consuming any red meat; *n* = 126)	8 week	1.6 (-21.6 to 24.7)	−2.5 (-13.6 to 8.7)	−21.2 (-43.5 to 1.1)	0.3994
	20 week	−5.9 (-29.6 to 17.8)	2.2 (-7.6 to 12.0)	−9.1 (-27.0 to 8.8)	
Alcohol (g/day) (consuming any alcohol; *n* = 126)	8 week	−0.4 (-2.1 to 1.3)	−2.4 (-5.0 to 0.2)	−3.4 (-8.4 to 1.5)	0.2361
	20 week	−1.8 (-4.2 to 0.6)	−1.4 (-4.5 to 1.7)	−1.1 (-6.1 to 3.9)	
Percentage of energy intake from alcohol (consuming any alcohol; *n* = 126)	8 week	−0.3 (-1.4 to 0.8)	−1.1 (-2.2 to 0.1)	−0.7 (-3.1 to 1.7)	0.4335
	20 week	−0.5 (-1.8 to 0.8)	−0.2 (-1.9 to 1.6)	0.4 (-2.3 to 3.1)	
Fruit serves/day (where baseline fruit consumption is less than recommended 2 serves/day; n = 93)	8 week	0.4 (0.2 to 0.6)	0.4 (0.1 to 0.7)	0.04 (-0.4 to 0.4)	0.5091
	20 week	0.4 (0.2 to 0.6)	0.6 (0.3 to 1.0)	0.3 (-0.2 to 0.7)	

Due to inadequate numbers, the impact of the intervention on cancer survivor or carer status could not be assessed separately. However, sensitivity analysis was undertaken to explore the effect of the intervention on the sample of cancer survivors only (*n* = 108), and is reported in Table 6. The adjusted mean difference between intervention and control at 8 weeks for daily step counts was 1998 (95 % CI: 707 to 3288), and at 20 weeks was 1402 (95 % CI: -379 to 3183) (*P* = 0.0111). The adjusted mean difference between groups on vegetable consumption at 8 weeks was 13.2 g per day (95 % CI: -12.9 to 39.3), and at 20 weeks was 39.2 g (95 % CI: 8.4 to 69.9) (*P* = 0.042). For BMI, the difference between groups at 8 weeks was -0.3 kg/m^2^ (95 % CI: -0.6 to -0.05), and at 20 weeks was -0.5 kg/m^2^ (95 % CI: -0.9 to 0.02) (*P* = 0.064). For weight, the adjusted between group difference at 8 weeks was -0.9 kg (95 % CI: -1.7 to -0.1), and at 20 weeks was -1.2 kg (95 % CI: -2.5 to 0.1) (*P* = 0.072).

**Table 6:** Mean difference in the physical activity, diet and body composition outcomes from baseline to 8 weeks and 20 weeks, and *p* value for the difference in change between treatment groups for cancer survivors only (n = 108)

		Mean change from baseline (95 % CI)		Adjusted mean difference (95 % CI)	Group-by-time
Outcome	Time from baseline	Control	Intervention		*P*-value
Mean daily steps (pedometer-assessed)	8 week	−1281 (-2349 to -214.2)	716.2 (-9.9 to 1442.4)	1997.6 (706.9 to 3288.4)	0.0108
	20 week	−1278 (-2587 to 30.8)	123.9 (-1084 to 1331.6)	1401.9 (-379.0 to 3182.8)	
Mean daily steps (with imputation of steps)	8 week	−1783 (-3200 to -366.2)	628.9 (-354.1 to 1611.9)	2411.9 (687.5 to 4136.4	0.0121
	20 week	−2220 (-3874 to -565.3)	327.0 (-1285 to 1939.1)	2546.6 (236.7 to 4856.5)	
Moderate-to-vigorous PA (minutes/week)	8 week	−1.8 (-33.4 to 29.7)	41.3 (-4.0 to 86.6)	43.2 (-12.0 to 98.4)	0.2064
	20 week	5.1 (-32.8 to 43.0)	−10.2 (-36.7 to 16.3)	−15.3 (-61.5 to 31.0)	
Resistance training (minutes/week)	8 week	18.6 (-0.4 to 37.5)	35.3 (14.8 to 55.8)	16.7 (-11.2 to 44.6)	0.403
	20 week	12.0 (-14.0 to 38.0)	24.5 (9.2 to 39.8)	12.5 (-17.7 to 42.7)	
Total sitting time on last WORK DAY, excluding sleep (minutes/day):	8 week	210.0 (-177.5 to 597.5)	3.5 (-138.7 to 145.7)	−206.5 (-619.3 to 206.2)	0.5880
	20 week	154.4 (-5.22 to 313.9)	135.0 (-122.1 to 392.2)	−19.3 (-322.0 to 283.3)	
Total sitting time on last NON-WORK DAY, excluding sleep (minutes/day):	8 week	125.5 (-137.3 to 388.3)	6.8 (-136.2 to 149.8)	−118.7 (-417.9 to 180.5)	0.1271
	20 week	−104.1 (-290.5 to 82.3)	82.3 (-78.3 to 242.9)	186.4 (-59.6 to 432.5)	
Fruit (excluding juice) (g/day)	8 week	−5.9 (-45.6 to 33.8)	37.5 (-7.2 to 82.1)	43.4 (-16.3 to 103.1)	0.1624
	20 week	−7.8 (-38.0 to 22.4)	56.9 (-5.4 to 119.2)	64.7 (-4.5 to 133.9)	
Vegetables (g/day)	8 week	5.6 (-12.3 to 23.5)	18.8 (-0.2 to 37.8)	13.2 (-12.9 to 39.3)	0.0422
	20 week	−7.4 (-27.2 to .12.4)	31.8 (8.2 to 55.3)	39.2 (8.4 to 69.9)	
Vegetables (serves/day)	8 week	0.1 (-0.2 to 0.3)	0.3 (-0.0 to 0.5)	0.2 (-0.2 to 0.5)	0.0422
	20 week	−0.1 (-0.4 to 0.2)	0.4 (0.1 to 0.7)	0.5 (0.1 to 0.9)	
Dietary fibre (g/1000 kJ)	8 week	0.1 (-0.03 to 0.3)	0.3 (0.1 to 0.4)	0.1 (-0.1 to 0.4)	0.2039
	20 week	−0.03 (-0.2 to 0.1)	0.2 (-0.00 to 0.4)	0.2 (-0.04 to 0.5)	
Energy (kJ/day)	8 week	−275 (-853 to 304)	−428 (-900 to 44)	−154 (-900 to 593)	0.7818
	20 week	101 (-1027 to 1229)	−336 (-868 to 195)	−437 (-1685 to 810)	
Total fat (g/day)	8 week	−3.5 (-10.7 to 3.7)	−7.4 (-12.4 to -2.4)	−3.8 (-12.6 to 4.9)	0.4710
	20 week	1.3 (-11.7 to 14.4)	−6.8 (-12.2 to -1.5)	−8.2 (-22.2 to 6.0)	
Saturated fat (g/day)	8 week	−1.6 (-4.3 to 1.0)	−3.5 (-5.6 to -1.4)	−1.8 (-5.2 to 1.5)	0.4082
	20 week	−0.00 (-4.7 to 4.7)	−3.0 (-5.0 to -1.0)	−3.0 (-8.1 to 2.1)	
Red meat (g/day)	8 week	12.8 (-0.3 to 25.9)	2.1 (-8.4 to 12.5)	−10.8 (-27.5 to 6.0)	0.4353
	20 week	4.6 (-10.0 to 19.2)	3.4 (-5.7 to 12.4)	−1.3 (-18.4 to 15.9)	
Processed meat (g/day)	8 week	−1.8 (-6.6 to 3.1)	0.3 (-2.2 to 2.7)	2.1 (-3.3 to 7.5)	0.7480
	20 week	−2.3 (-9.9 to 5.3)	0.1 (-3.5 to 3.7)	2.4 (-6.0 to 10.8)	
Alcohol (g/day)	8 week	−0.1 (-1.8 to 1.5)	−1.1 (-3.1 to 1.0)	−0.9 (-3.5 to 1.7)	0.5974
	20 week	−1.2 (-3.5 to 1.1)	−1.3 (-3.7 to 1.1)	−0.1 (-3.4 to 3.2)	
% of energy provided by alcohol (%)	8 week	−0.2 (-1.2 to 0.9)	−0.4 (-1.2 to 0.4)	−0.2 (-1.5 to 1.1)	0.9382
	20 week	−0.4 (-1.6 to 0.9)	−0.4 (-1.5 to 0.6)	−0.1 (-1.7 to 1.5)	
Weight (kgs)	8 week	0.1 (-0.5 to 0.7)	−0.8 (-1.4 to -0.3)	−0.9 (-1.7 to -0.1)	0.0723
	20 week	−0.1 (-1.0, 0.7)	−1.3 (-2.3 to -0.3)	−1.2 (-2.5 to 0.1)	
BMI (kg/m^2^)	8 week	0.04 (-0.2 to 0.3)	−0.3 (-0.5 to -0.1)	−0.3 (-0.6 to -0.05)	0.0637
	20 week	−0.03 (-0.3 to 0.3)	−0.5 (-0.9 to -0.1)	−0. 5 (-0.9 to 0.02)	
Waist circumference (cm)	8 week	−2.0 (-4.7 to 0.8)	−2.9 (-5.3 to -0.4)	−0.9 (-4.6 to 2.8)	0.6287
	20 week	−2.3 (-4.6 to 0.1)	−1.4 (-5.6 to 2.9)	0.9 (-4.0 to 5.7)	

## Discussion

### Statement of principal findings

The primary aim of this paper was to report the effects of a theory-based, group-delivered, face-to-face, multiple health behavior change intervention (ENRICH) on behavioral outcomes among a mixed group of cancer survivors and carers. The ENRICH multiple health behavior intervention was effective for improving pedometer-assessed PA, weight, and subsequently body mass index, and vegetable consumption. Achieving improvements in at least one component of both diet and PA behaviors is an important finding, and has demonstrated potential to improve health outcomes, such as body composition and chronic disease risk [58, 59].

The improvements in pedometer step counts are lower than the results reported in reviews of pedometer interventions with adults [45, 60, 61]. While the increases in the intervention group were small, the control group decreased steps by more than 1000 steps. This difference between groups of 2000 steps per day may be clinically important, as an increase of 2000 steps has been associated with decreased blood pressure, BMI, and an 8 % decrease in cardiovascular event rate [45, 62]. Both groups successfully increased their time spent in moderate and vigorous PA and resistance training, however these changes were not significant and might reflect that simply enrolling in a lifestyle behavior modification trial is sufficient to stimulate change. Imputation of step count values for swimming, cycling, and resistance training, had a significant effect between the two groups with the mean difference increasing by approximately 1000 steps (from 2000 to 3000 steps). Whilst it did not change interpretation of the results, it amplified the difference between the two groups and reflects that this target group do participate in activities not captured by pedometry.

Both groups in the current trial showed encouraging (non-significant) trends in regards to fruit, alcohol and fat consumption; similar to the FRESH START intervention which reported significantly improved lifestyle behaviors over 12 months [29]. The only significant dietary impact in the present study was the increase in vegetable consumption in the intervention group by 0.4 serve (32 g) at 20 weeks, similar to an 11-session telephone counseling intervention that targeted colorectal cancer survivors [30]. Our findings are of a similar magnitude to those found amongst breast and prostate cancer survivors who reported a difference of 0.5 serves per day of combined fruit and vegetable intake after a 12-month intervention with rigorous dietary goals [29]. Although this change is small, increases of one serve of vegetables per day have been associated with a 5 % reduction in all-cause mortality [63], and a 5 % reduction in ischaemic stroke risk and 10 % reduction in ischaemic heart disease [64]. While other studies have reported significant effects on fat consumption [30], intervention participants in the current study reported non-significant decreases in fat and energy intake. Diet was a secondary outcome, so the study may have been under-powered to detect small changes to all aspects of diet. It is also important to note that the FFQ is not designed to assess small changes in diet, and being a self-report measure, there may also be an association with reporting bias. The magnitude of change for participants in both groups who were not already meeting recommended two serves of fruit per day at baseline was higher than the total sample, which may indicate possible ceiling effects.

Despite ENRICH not being designed or promoted as a weight loss intervention, the intervention group reported significant decreases in weight and BMI at both 8- and 20-weeks. Although both groups reported decreases in waist circumference, the group-by-time effect were not statistically significant. Significant changes to BMI were also reported in the Australian CanCHANGE intervention with BMI decreasing by 0.9 at 12 month follow-up [30]. Intervention participants decreased weight by 1.9 % at 8 weeks, and 2.2 % at 20 weeks. Whilst a 5 % reduction in weight is considered to be a clinically significant threshold [65], health improvements have been noted for smaller reductions in weight (at 2 %) and waist circumference (at 4 cm) [66, 67]. Sub-group analyses showed that the intervention appeared to have a stronger effect on the step counts, weight, and BMI, of overweight or obese participants when compared to participants who were underweight or healthy weight at baseline. It was encouraging that overweight and obese participants were able to undertake increased PA after the face-to-face intervention had finished.

Exploratory sub-group analyses of cancer survivors revealed some possible differences between cancer survivors’ and carers’ response to the intervention. Differences between groups over time remained significant for mean daily step counts and vegetable consumption. However, the magnitude of change among cancer survivors was smaller than the increases observed in the total sample. Reductions in weight and BMI were reported among cancer survivors, however these group-by-time changes were not significant. There are few trials that include carers in behavior change interventions to compare these findings to, however the results of this trial are promising for carers.

As a simultaneously delivered multiple health behavior change program, the ENRICH intervention was designed so that participants were encouraged to reflect how their current behavior corresponded to current recommendations and then select behaviors they wanted to change. It is therefore unrealistic that participants would make positive changes in every behavior, making assessment of changes at the group level challenging. In addition, improvements in one area may have resulted in other compensatory behaviors (e.g., success in achieving a self-monitored step count goal may have led to increased energy consumption). Other research has reported an association between increased PA levels and increased fat intake [68]. Most previous multiple health behavior interventions have been of longer duration (6-12 months) and greater intensity [29, 36, 69] than the intervention presented here. It was encouraging that behavior change was achieved with a relatively low dose, short duration intervention (total 12 h of contact), and change was sustained over the short-term.

### Strengths and weaknesses of the study

Targeting cancer survivors of mixed diagnoses and carers is a novel aspect of the program, however, this heterogeneity prevented sub-group analyses by sex, cancer type, or carer status. Given the pragmatic, community-based recruitment methods used in this trial, participants were not broadly representative of the cancer survivor population. Similar to many behavior change trials, breast cancer survivors and participants with high socioeconomic status were over-represented and men were under-represented in our sample [6, 70]. The majority of participants were at least 3 to 5 years post-diagnosis at baseline. In addition, participants were likely to be more interested (and potentially motivated) to make lifestyle changes. Wait-list control participants’ awareness that they were in a diet and PA study and receipt of (sealed) pedometers to record steps at each time-point may have influenced their PA behavior [45, 60, 71]. Using accelerometers to measure PA and sitting time would provide an objective measure of the duration and intensity of activity.

Although the drop-out rate after baseline data collection was high (19.5 % of the total sample), we successfully retained 75 % of intervention participants who attended at least one intervention session. A wait-list control group was used to enhance recruitment and to meet ethics requirements regarding provision of care to participants. However it appears that the wait-list control group was not acceptable to many participants due to the long wait before attending, or difficulty attending on the specific dates (e.g., due to pre-planned holidays), or due to a change in their circumstances (e.g., return to work). Recent data have confirmed that offering participation in the intervention at completion of the study does not compensate completely for the disappointment of being assigned to the control group [72]. Our trial duration was less than 6 months and wait-list control participants were expected to provide three measures of 7-day pedometry, and complete three separate surveys before receiving any support or assistance. These findings have implications for researchers designing behavior change trials, and especially for trials that involve longer term follow-ups. There remains a need to consider alternate trial designs (e.g., attention control designs) to reduce the impact of drop-out due to study group preferences. Intervention attrition was similar to other face-to-face intervention trials with a 12-month follow-up [73, 74]. The most common reasons for attrition and drop out related to pragmatic difficulties with program scheduling (location and/or time), changed work or personal circumstances that prevent attendance, or illness or injury [74]. While illness or injury was reported as a reason for withdrawal, it was unrelated to the intervention or research study. We acknowledge that the attrition has resulted in missing data over time, however the use of linear mixed models has been shown to be robust when missing data depends on baseline values [75].

### Unanswered questions and future research

There remains a need to identify more programs that can improve lifestyle behaviors among cancer survivors and carers. As with other trials, this study was not able to successfully recruit participants most at-risk. There remains a need to promote programs that are appealing to cancer survivors earlier in their cancer trajectory in order to improve their health outcomes. Interventions that appeal to men and those who are socially disadvantaged, and are accessible regardless of geographic location are needed. Despite participants in this trial being interested and motivated to change behavior, retention of participants was a significant issue. The intervention was effective for the group recruited, however the study was not able to detect the separate impact of the intervention on specific cancer types, on carers, or on men. Future trials should assess intervention effects separately for cancer survivors and carers. The magnitude of behavior changes achieved in this trial was similar to other interventions delivered through different channels (such as telephone) and to those of longer duration. This offers reassurance to those considering programs in this target group. A range of lifestyle interventions is likely to be required to meet the diverse needs of cancer survivors and carers in the future. Future multiple health behavior change trials will benefit from using objective measures of sedentary behavior and PA, such as inclinometers and accelerometers, and using dietary measures that are sensitive to small changes. Trials that assess maintenance of behavior change over the longer-term are still required.

## Conclusions

Cancer survivors and carers can participate in a unique theoretically-based program targeting aerobic activity, resistance training, and healthy diet components. Significant improvements to objectively measured steps, weight, body mass index, and vegetable consumption were sustained for three months after completion of the intervention. The magnitude of behavior change was significant and clinically relevant, and likely to result in health improvements. ENRICH has subsequently been adopted as a Cancer Council NSW program and is being delivered state-wide.

## Abbreviations

ENRICH: Exercise and Nutrition Routine Improving Cancer Health
PA: Physical activity
RCT: Randomized controlled trial
FFQ: Food frequency questionnaire
BMI: Body mass index
